# Cefiderocol in Children with Hematologic Malignancies—The Multicenter Retrospective Experience of the Infection Working Group of the Italian Pediatric Hematology and Oncology Association (AIEOP)

**DOI:** 10.3390/jcm15083100

**Published:** 2026-04-18

**Authors:** Paola Muggeo, Federica Galaverna, Lorenzo Chiusaroli, Katia Perruccio, Paola Coccia, Francesco Baccelli, Emilia Boccieri, Chiara Rosignoli, Francesco De Leonardis, Nicola Santoro, Simone Cesaro

**Affiliations:** 1 U.O.C. Pediatria ad Indirizzo Oncoematologico, Azienda Ospedaliero Universitaria Consorziale Policlinico, 70124 Bari, Italy; paola.muggeo@policlinico.ba.it (P.M.); fdl111@libero.it (F.D.L.); nico.santoro1956@libero.it (N.S.); 2 Department of Pediatric Oncology, Hematology, Cell and Gene Therapy, IRCCS Bambino Gesù Pediatric Hospital, 00165 Rome, Italy; federica.galaverna@opbg.net (F.G.); emilia.boccieri@opbg.net (E.B.); chiara.rosignoli@opbg.net (C.R.); 3 Division of Pediatric Infectious Diseases, Department for Women’s and Children’s Health, University of Padua, 35128 Padua, Italy; lorenzo.chiusaroli@phd.unipd.it; 4 Oncoematologia Pediatrica, Ospedale Santa Maria della Misericordia, 06129 Perugia, Italy; katia.perruccio@ospedale.perugia.it; 5 Oncoematologia Pediatrica, Presidio Ospedaliero “G. Salesi”, 60123 Ancona, Italy; paola.coccia@ospedaliriuniti.marche.it; 6 Dipartimento di Scienze Mediche e Chirurgiche (DIMEC), University of Bologna, 40126 Bologna, Italy; francesco.baccelli2@unibo.studio.it; 7 Pediatric Hematology and Oncology, IRCCS Azienda Ospedaliero-Universitaria di Bologna, 40126 Bologna, Italy; 8 Pediatric Hematology Oncology, Department of Mother and Child, Azienda Ospedaliera Univeritaria Integrata, 37126 Verona, Italy

**Keywords:** cefiderocol, children, cancer, pediatric hematology, hematopoietic stem cell transplantation

## Abstract

**Background/Objectives**: Immunocompromised children undergoing chemotherapy or allogeneic hematopoietic stem cell transplantation (HSCT) for hematologic disorders face a high risk of serious, life-threatening infections caused by multidrug-resistant (MDR) bacteria. Cefiderocol is a novel siderophore cephalosporin, indicated for use in adult patients with MDR Gram-negative infections. Clinical data in immunocompromised children are limited. To report a multicenter real-life experience from the Infection Working Group of the Italian Pediatric Hematology and Oncology Association (IWG-AIEOP) on the use of cefiderocol in treating pediatric onco-hematologic patients with severe, high-risk infections. **Methods**: Multicenter retrospective collection of infectious episodes treated with cefiderocol, from January 2021 to December 2024, in patients 18 years or younger, after treatment for malignancies or undergoing HSCT in the AIEOP network, part of a prospective, observational study on the etiology and outcome of febrile episodes among 24 AIEOP centers (code NCT06419426). **Results**: Fifteen episodes of MDR, life-threatening Gram-negative infections treated with cefiderocol in 13 pediatric onco-hematologic patients were collected. There were eight males and five females, mainly affected by acute leukemia (six lymphoblastic and four myeloid, three other hematologic malignancies). The median age was 11.1 years (range 1–17.4 years), and the median weight was 37.8 kg (range 8–65). Bloodstream infection occurred in 10 of 15 episodes. *Pseudomonas aeruginosa*, *Klebsiella pneumoniae*, and *Stenotrophomonas maltophilia* were isolated in 11, 3, and 1 episodes, respectively. Notably, 11 of 15 isolated pathogens carried a metallo-beta-lactamase (MBL) gene (Verona integron-encoded, VIM, *n* = 10; New Delhi, NDM, *n* = 1). All patients achieved infection resolution and were alive and infection-free 90 days after infection onset. **Conclusions**: Cefiderocol was well tolerated and showed encouraging, favorable clinical outcomes, without serious adverse effects.

## 1. Introduction

The rise of multidrug-resistant (MDR) organisms is a major global concern, particularly in immunocompromised patients, such as those with cancer or undergoing hematopoietic stem cell transplantation (HSCT) [1]. Infections caused by MDR Gram-negative bacteria are associated with increased morbidity and mortality, as there is a limited pipeline of effective antibiotics, and carbapenemase-producing strains can become resistant even to the best available treatments [2,3]. The concern is even greater in children under 18 years of age due to the limited availability of newly licensed adult drugs for the pediatric population, which are often accessible only on an individual, off-label basis [4]. Recently, the 2020 European Conference on Infections in Leukaemia 8 (ECIL8) panel of experts highlighted the growing problem of MDR bacteria worldwide and identified the need for new antibiotics as a critical gap to be addressed [5].

Cefiderocol is a novel siderophore cephalosporin indicated for adult patients with limited therapeutic options against MDR Gram-negative bacteria [6]. According to the Infectious Diseases Society of America (IDSA) guidelines, cefiderocol is recommended for carbapenem-resistant (CR) *Enterobacterales*, CR *Acinetobacter* (*A.*) *baumannii*, difficult-to-treat *Pseudomonas* (*P.*) *aeruginosa*, and *Stenotrophomonas* (*S.*) *maltophilia* [7]. Notably, cefiderocol is stable against hydrolysis by many beta-lactamases [8,9], and it is the only beta-lactam antibiotic with in vitro activity against both serine-carbapenemases and metallo-carbapenemases as well as other broad-spectrum serine beta-lactamases, such as AmpC chromosomal class C enzymes [10]. Cefiderocol susceptibility rates in both Europe and the United States exceed 90% for a range of aerobic Gram-negative isolates collected from adult patients [10,11,12], although lower activity has been reported in areas with certain metallo-beta-lactamase (MBL) prevalence, such as New Delhi metallo-beta-lactamase (NDM) [10,13].

Robust data on the use of cefiderocol for the treatment of multidrug-resistant, carbapenem-resistant, Gram-negative bacterial infections in adult patients have been published, including studies involving immunocompromised patients [14,15]. Recently, the international multicenter “PROVE” chart review study confirmed the real-world efficacy and safety of cefiderocol in adult patients with serious Gram-negative infections; notably, 60% of this cohort was in the intensive care unit, and almost 50% were receiving organ support [16].

In contrast, the use of cefiderocol in pediatric patients has been reported only in single case reports [17,18,19,20,21] or in small, retrospective, single-center case series [22]. Moreover, data on pediatric hematology–oncology patients are limited to a few single case reports [23,24]. An international, open-label, multicenter phase II trial reported good safety and tolerability of cefiderocol in 53 pediatric patients (median age 73.5 months, range 3–215) with aerobic Gram-negative infections, showing a good pharmacokinetic profile, no discontinuations due to toxicity, and similar pharmacokinetics when comparing single versus multiple doses [25].

We report a multicenter real-life experience from the Infection Working Group of the Italian Pediatric Hematology and Oncology Association (IWG-AIEOP) on the use of cefiderocol in treating onco-hematological children and adolescents with severe high-risk infections, providing information on efficacy and tolerability in the off-label setting of onco-hematological pediatric patients.

## 2. Materials and Methods

The IWG-AIEOP retrospectively assessed the safety and efficacy of cefiderocol in the treatment of bloodstream infections (BSIs) caused by MDR organisms in febrile onco-hematological children and adolescents (age range 0–18 years). The BSI episodes analyzed in this study were part of a national, prospective, observational study on the etiology and outcome of febrile episodes conducted from January 2021 to December 2024 among 24 centers belonging to AIEOP (code NCT06419426). Cefiderocol was administered in 17 febrile episodes for 15 patients. Two patients were excluded because after 24 h cefiderocol was replaced with more targeted antibiotic therapy. The primary endpoint was to describe the safety and toxicity profile of cefiderocol used as first-line or salvage treatment, while the secondary endpoint was the outcome of the infectious episode, defined as complete response/resolution, improved, stable, or worsening, at 30 and 90 days from the start of BSI. Data collection was conducted using a paper case report form that required anonymized demographic, clinical, and microbiological characteristics of the infectious episode. Parent and/or patient informed consent was obtained, and data were handled in accordance with the Italian regulations for patient confidentiality and good clinical practice. Severe neutropenia and very severe neutropenia were defined as an absolute neutrophil count (ANC) at the onset of the febrile episode of less than 0.5 × 10^9^/L and 0.1 × 10^9^/L, respectively. In accordance with standardized international definitions [26], MDR and extensively drug-resistant (XDR) Gram-negative bacteria were defined as pathogens that are non-susceptible to at least one agent in three or more different antimicrobial classes or to at least one agent in all but two or fewer antimicrobial categories, respectively. Susceptibility criteria for antimicrobial agents were based on current CLSI, EUCAST, and FDA guidelines, supplemented by Expert Group opinions. Surveillance rectal swabs for MDR/XDR bacteria were collected at admission and weekly thereafter during hospitalization as part of the ongoing Italian program for MDR/XDR bacteria surveillance according to Italian Ministerial recommendations [27]. Patients were considered colonized if a rectal swab was positive for MDR/XDR bacteria up to three months prior to the infectious episode. The prescription of cefiderocol was determined by the local treating physician, based on a named-patient off-label authorization, according to best practice recommendations or center policy for the treatment of Gram-negative MDR, MBL-producer, or difficult-to-treat Gram-negative BSI [7]. Organ toxicity during infection was assessed using the Common Terminology Criteria for Adverse Events (CTCAE) scale, version 4.0. Descriptive statistics (means, medians, percentages) were used to report the variables of interest in aggregate.

## 3. Results

Cefiderocol was administered for 15 febrile episodes caused by MDR bacteria in 13 patients with hematological malignancies under treatment; 2 patients received two courses of cefiderocol due to a second episode of MDR bacteremia. The median age was 8.5 years (range 1–17.4 years), and the median weight was 37.8 kg (range 8–65 kg). Clinical characteristics are reported in Table 1.

All patients had severe neutropenia (ANC < 500/mmc). The median duration of neutropenia was 19 days (IQR = 10–60 days), while the median interval between the onset of neutropenia and Gram-negative infection was 8 days (IQR = 5–20 days). An ANC < 100/mmc was present in 13 of 15 episodes, with a median total duration of 17 days (IQR = 11–28 days). Six episodes occurred following HSCT, while nine episodes were registered during first-line treatment (*n* = 5) or salvage treatment (*n* = 4) for hematological malignancies. The infectious episodes occurred a median of 27 days (IQR = 11–104 days) following the last hematological treatment. Prior intestinal colonization was present in 10 out of 13 children: *P. aeruginosa* XDR Verona integron-encoded (VIM)+ was detected in 5 patients, *P. aeruginosa* XDR carbapenem-resistant organism (CRO) carbapenemase-producing organism (CPO) in 2 patients, and *Klebsiella (K.) pneumoniae* XDR NDM+, *K. pneumoniae* XDR ESBL+, *Enterobacter cloacae* complex VIM+ in 1 patient each. Additionally, one patient colonized by *Enterobacter cloacae* complex MBL VIM+ subsequently developed bacteremia due to *K. pneumoniae* VIM+. Microbiological characteristics of infectious episodes are detailed in Table 2.

In our cohort, BSI represented the main indication to use cefiderocol (*n* = 10), with 4 out of 10 episodes presenting both BSI and localized infection (one pneumonia, one urethritis, two soft tissue infection—perianal lesion, cheek tissue). Abdominal abscess, pneumonia, and urethritis without bacteremia were responsible for one case each, respectively, with the isolation of MDR bacteria in urethral swab, bronchoalveolar lavage, and stools, respectively. No pathogen except MDR colonization was identified in the remaining two cases. BSI isolated the same MDR bacteria detected in previous colonization in 8 out of 10 colonized patients. Two of them developed a second episode of BSI during subsequent chemotherapy-induced neutropenia, after 45 and 120 days from the first one, respectively. Moreover, microbiological isolates detected *P. aeruginosa*, *K. pneumoniae*, and *S. maltophilia* were isolated in 11, 3, and 1 episodes, respectively; the relapsing 2 out of 15 infectious episodes isolated the same MDR *P. aeruginosa* that was responsible for the first infection, and both patients carried *P. aeruginosa* intestinal colonization. Intriguingly, one patient experienced MDR bacteremia during third-line salvage treatment for post-HSCT relapsing ALL, after one year of hematological remission with no detection of MDR colonization, while another patient, colonized by *E. cloacae complex* VIM+, developed BSI due to *K. pneumoniae* VIM+. The microbiologically documented infections without bacteremia included: one pneumonia due to *S*. *maltophilia*, trimethoprim/sulfamethoxazole-resistant isolated by a bronchoalveolar lavage; one urethritis due to *P. aeruginosa* identified by urethral swab; and an abdominal abscess due to *P. aeruginosa*. Interestingly, *P. aeruginosa* was responsible for three out of four cases of both BSI and localized infection (one pneumonia, two soft tissue, cheek and perianal abscess), while *K. pneumoniae* was associated with BSI and urethritis. The MBL gene was detected in 11/15 isolates: VIM gene in 10 cases (one *E. cloacae*, and 7 *P. aeruginosa* responsible for one and nine episodes, respectively), and NDM gene in 1 case (*K. pneumoniae*). Patients received cefiderocol at a dose of 60 mg/kg/dose every 8 h, with a maximum dosage of 2 g three times daily for children weighing 34 kg or more, according to the phase II PEDI-CEFI pediatric trial [25]. Cefiderocol was started at a median of 24 h (range 2 h–16 days) from the onset of infection, as first-line treatment in nine episodes, or added following the blood culture results or information on pathogen susceptibility in six cases. All patients received cefiderocol associated with other antibiotic treatment (amikacin *n* = 4, meropenem *n* = 7, colistin *n* = 8, fosfomycin *n* = 7, ceftazidime-avibactam *n* = 3, aztreonam *n* = 1, tigecycline *n* = 2, levofloxacin = 1 case, respectively), with the most used combinations being cefiderocol + colistin in eight cases and cefiderocol + colistin + fosfomycin in five cases respectively. The median treatment duration was 15 days (range 3–41). Table 3 reports the clinical characteristics of infectious episodes.

Cefiderocol was well tolerated. Importantly, no patient needed drug discontinuation due to adverse events, and the assessment of organ toxicity did not report any serious adverse effects. Two patients presented red wine urine syndrome, which resolved after cefiderocol discontinuation. Fever > 38 °C was the most common clinical sign at the onset of infection (14/15 episodes), and concomitant organ dysfunction was present in four cases (two cases of hypotension and two of hypoxemia). Oxygen support and vasoactive amine administration were necessary in five and two episodes, respectively, while one patient was admitted to the intensive care unit due to the development of acute respiratory distress. Median time from infection onset to neutrophil recovery was 10 days (IQR 19-6). Eleven out of fifteen episodes were resolved at 30 days after infection onset; improving clinical conditions with ongoing antibiotic treatment were reported in four cases. All patients eventually achieved infection resolution and were alive and infection-free at 90 days after infection onset. However, two patients with persistent MDR Gram-negative intestinal colonization experienced a second febrile episode during subsequent treatment-induced neutropenia. During later follow-up, two deaths were recorded due to relapse of the hematological disease, 6 and 18 months after the infectious episode, respectively. Detailed features for each episode are presented in Table 4.

## 4. Discussion

We report real-life experience with cefiderocol use in children and adolescents with hematological malignancies and life-threatening infections caused by MDR Gram-negative bacteria. While substantial data are available for adult patients [28], evidence in the pediatric population remains limited.

The bacteria isolated from the analyzed patients belonged to the so-called “critical” group (CR *A. baumanii* and *Enterobacterales*) and “high-risk” group (CR *P. aeruginosa*, Table 2) according to the “World Health Organization (WHO) Bacterial Priority Pathogen List” [1]. In the recently published WHO Pediatric Drug Optimization “priority list”, which identifies pipeline or newly approved antibiotics without pediatric indications as priorities for further investigation and development in children, cefiderocol is one of four antibiotics [29]. This is due to its important activity against MBL-producing *Enterobacterales*, for which few other options exist and which are the most prevalent strains of CRO in many areas.

According to the IDSA guidelines [7], cefiderocol is recommended for the treatment of infections caused by carbapenem-resistant *Enterobacterales* (CRE), difficult-to-treat *P. aeruginosa* (DTR-PA), carbapenem-resistant *A. baumannii* (CRAB), and *S. maltophilia*. However, the Food and Drug Administration (FDA) and the European Medicines Agency (EMA) have not yet approved its use in the pediatric population [30,31].

Very limited data have been reported on the use of cefiderocol in patients under 18 years, mainly as case reports [17,18,19,20,21,23,24] or case series [22]. Schmid et al. reported 18 episodes of severe Gram-negative infections in 14 patients with different diseases, 8 of whom were immunocompromised. Four of these patients had hematological malignancies or had undergone HSCT, comparable to the thirteen patients in our case series. In the Schmid series, cefiderocol was well tolerated, with a low mortality rate compared to adults, and a 30-day mortality rate of 10% among the 10 children with severe infections.

**Figure 1:**
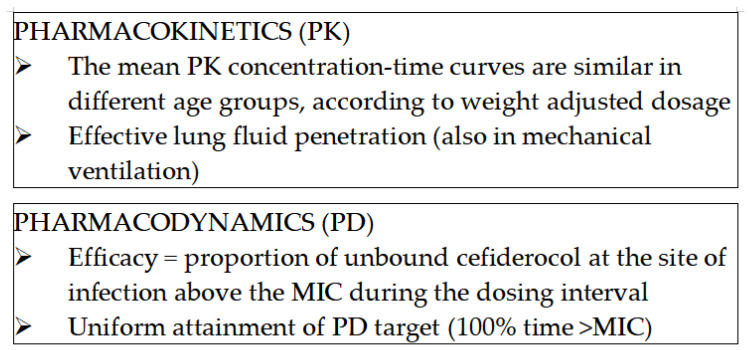
Key points of the pharmacokinetics and pharmacodynamics of cefiderocol.

Currently, no standardized pediatric dosing guidelines for cefiderocol are available [32]. To address this gap, three clinical trials evaluated the safety, efficacy, and appropriate dosing of cefiderocol in children. The results confirmed that cefiderocol administered at a dose of 60 mg/kg every 8 h, with a maximum dosage of 2 g three times daily for children weighing 34 kg or more achieved adequate steady-state plasma concentrations and was well tolerated [25]. The same dosage was confirmed in a recently published consensus-based document [33], which suggested optimal dosing for beta-lactam agents used to treat MDR Gram-negative infections in pediatric patients. Data on the pharmacokinetics and pharmacodynamics of cefiderocol in children are reported in the phase II PEDI-CEFI study [25], which provides efficacious pharmacokinetic curves and bioavailability when administering multiple doses according to weight as described. In Figure 1, we present the key points of the pharmacokinetics and pharmacodynamics of cefiderocol.

In the present experience, cefiderocol was well tolerated. Notably, only two patients experienced mild, self-limiting adverse events (AEs) characterized by red-colored urine, often referred to as “red wine urine syndrome.” The findings, likely observed after administration of an iron-containing product, are consistent with previous reports [34]. They were also reported in one patient in the case series by Schmid et al. [22] as well. As with the patients previously described, the two patients in our series had received iron-containing products, such as packed red blood cells, as expected in patients undergoing treatment for cancer or HSCT. In adults, post-marketing surveillance confirmed the observation of reversible chromaturia and raised concerns about potential nephrotoxic effects, including tubulointerstitial nephritis and acute kidney injury, as documented in the U.S. FDA Adverse Event Reporting System [35]. In contrast, a recent real-world study conducted in Spain involving 314 adult patients treated with cefiderocol reported only seven AEs, with three classified as serious AEs (SAEs) [36]. These findings align with those of a recent systematic review and meta-analysis, which found no significant differences in the incidence of AEs or SAEs between cefiderocol and other beta-lactam antibiotics, including meropenem, polymyxin B, and imipenem-cilastatin [37].

Given the limited data in the pediatric literature supporting the real-life use of cefiderocol, we emphasize the importance of our observation of only mild adverse events, even within such a vulnerable cohort of pediatric patients with onco-hematological malignancies.

Moreover, optimizing infection and support treatment for immunocompromised children represents one of the greatest challenges for the treating physicians. In most patients, conventional chemotherapy still represents the backbone of first-line cancer treatment, and unavoidable treatment-related toxicity and immune suppression often lead to a fragile clinical condition. A damaged immune system results in impaired immune response to pathogens, thus leading to increased risk of infection dissemination and end-organ damage.

In this context, the use of biomarkers could help predict the severity of infection. Recently, the role of new inflammatory biomarkers such as butyrylcholinesterase has emerged. According to the literature, low levels of this enzyme are associated with an increased risk of developing wound infections in patients undergoing surgery [38]. Moreover, in critically ill children with bloodstream infections, butyrylcholinesterase was recently reported as an independent risk factor for all-cause mortality [39].

Therefore, immunocompromised children experiencing infections due to MDR organisms represent a crucial target for effective new antimicrobial agents. Our report confirmed that cefiderocol was effective in rescuing these frail children with MDR Gram-negative disseminated infection and was able to compensate an impaired immune system. On one hand, cancer treatment in the era of immune and target therapy aims to reduce treatment-induced complications preserving and even improving efficacy; on the other hand, antibiotic efficacy has become even more crucial in preserving patient conditions.

In our patient series, cefiderocol was always used in combination with other antimicrobial agents given the high risk of severe complications during MDR Gram-negative infections in neutropenic, immunocompromised patients and as suggested to enhance synergistic efficacy [40].

The use of cefiderocol in combination therapy could be a limitation of our study. However, the true impact of cefiderocol in combination and its synergism with other antimicrobial agents is still under investigation [41,42], and the emergence of resistance has already been reported in the literature [43,44], emphasizing the need for strict adherence to recommendations and avoidance of empirical use [28]. Interestingly, a promising approach to overcoming the emergence of resistance to cefiderocol was recently reported: zidebactam was identified as an adjuvant that synergistically enhances the activity of cefiderocol against resistant strains in vitro [45].

Notably, 11 successful treatments of MBL-producing Gram-negative bacterial infectious episodes have been reported, highlighting the specific efficacy of cefiderocol in this critically resistant cohort. Moreover, ceftazidime/avibactam does not show activity against VIM-MBL without aztreonam combination [46]; therefore, cefiderocol use should be reserved for patients in whom such a resistance pattern has been demonstrated or is highly suspected based on local epidemiology.

Two patients presented with recurrent bacteremia. These episodes, which occurred after discontinuation of antibiotic treatment, could be attributed to inadequate drug control. However, additional factors should also be taken into account. A positive rectal swab for CRO after previous negative results is common in the clinical and microbiological monitoring of patients at high risk for intestinal colonization by MDR Gram-negative bacteria, such as immunocompromised individuals receiving treatment for hematological malignancies. The persistence of risk factors combined with prior CRO colonization greatly increases the likelihood of colonization and bacteremia from MDR bacteria. A colonized patient should be considered as such for life [47]; however, once immunosuppression ends, a favorable microbiota can control the emergence of MDR bacterial flora. Antibiotic treatments for intestinal decolonization used in previous years are not currently recommended [48]. We believe that in such high-risk patient settings, there is concern regarding the persistence, or even worsening, of risk factors, which exposes patients to relapse of colonization and subsequently to serious, life-threatening infection. We cannot exclude a source control problem; in this regard, fecal microbiota transplantation as a pre-HSCT infection control strategy appears promising in cases of MDR Gram-negative intestinal colonization [49].

A controversial issue in cefiderocol treatment is susceptibility testing. The iron-binding property of cefiderocol makes accurate and reproducible antimicrobial susceptibility testing (AST) complex due to the requirement for iron-depleted media for culture, which lack reproducibility. According to the literature [50], commercial AST methods (broth microdilution, disc diffusion, and gradient diffusion tests) often exhibit high major error rates and should therefore be used with caution. In our case series, likely due to the lack of a standardized and validated method, AST was performed in only four patients (Table 4), using broth microdilution in one patient and disc diffusion test in two, which demonstrated the sensitivity to cefiderocol of the cultured bacteria. All infectious episodes reported in our series were caused by MDR bacteria according to standard international definitions [26]. For each episode, the sensitivity to either cefiderocol or another antibiotic used in combination with cefiderocol was reported (Table 4). In these patients, the use of combination therapy is crucial to save lives.

Cefiderocol is classified as a “reserve” compound according to the AWaRe classification [51]. This aims to limit empiric use to prevent the emergence of cefiderocol resistance, which severely threatens outcomes in critical infections caused by MDR Gram-negative bacteria. The field of antimicrobial resistance is highly dynamic; close monitoring of local epidemiology is warranted.

## 5. Conclusions

Despite the limitation of a small sample size, this multicenter experience suggests that cefiderocol is a safe and effective therapeutic option for children and adolescents with hemato-oncological conditions facing life-threatening infections caused by multidrug-resistant Gram-negative bacteria, particularly those producing metallo-β-lactamases (MBL). The introduction of novel antimicrobials in high-risk pediatric populations with limited treatment alternatives is warranted; however, close monitoring for the emergence of resistance remains essential. Prospective pediatric studies are needed to further evaluate the efficacy and safety of cefiderocol in this vulnerable group.

## Figures and Tables

**Table 1:** Clinical characteristics of patients.

Patients, *n*	13
Age, median (years)	8.5
range	1–17.4
Male/female	8/5
Underlying disease	
ALL	6
AML	4
Others ^a^	3
Treatment phase of hematological disease	
1st line chemotherapy	4
2nd or 3rd line chemotherapy	4 ^b^
Allogeneic SCT	5
Neutropenia (ANC < 100) (days)	
median (IQR)	17 (28–11)
Antibiotic prophylaxis	11/13
Time from treatment to onset of infection (days) median (IQR)	27 (104–11)
Previous intestinal MDR colonization	10/13

ALL: acute lymphoblastic leukemia; AML: acute myeloid leukemia; HSCT: hematopoietic stem cell transplantation; ^a^ including 2 myelodysplastic syndromes, 1 hemophagocytic lymphohistiocytosis; ^b^ one patient had a relapse of the hematological disease 1 year following HSCT for AML.

**Table 2:** Isolated carbapenem-resistant Gram-negative bacteria.

Episodes (*n* = 15)	Total	Intestinal	Bacteremia	Localized Infection	
		Colonization			
				nB	B
Isolated MDR Gram-Negative Bacteria					
*P. aeruginosa XDR VIM+*	9	7	6	1	2
*P. aeruginosa XDR CRO no CPO*	2	2	1	1	1
*K. pneumoniae XDR ESBL+*	1	1	1	0	0
*K. pneumoniae XDR NDM+*	1	1	1	0	1
*K. pneumoniae XDR VIM+*	1	0	1	0	0
*Enterobacter cloacae complex VIM+* ^a^	1	1	-	-	-
*S. maltophilia*	1	0	0	1	0

^a^ intestinal colonization due to VIM+ bacteria in a patient showing subsequent bloodstream infection due to VIM+ *K. pneumoniae*; MDR Gram-negative: multidrug-resistant Gram-negative; XDR extensively drug-resistant; NDM New Delhi metallo-beta-lactamase; VIM Verona integron-encoded metallo-beta-lactamase; nB: not bacteremic; B: bacteremic.

**Table 3:** Characteristics of infectious episodes.

Infectious episodes, *n*	15
Bacteremia	10
Site of infection ^a^	
bloodstream	10
lung	2
abdomen	1
urethra	2
soft tissue (cheek, perianal)	2
Type of infection	
non-complicated BSI	6
complicated BSI:	4
BSI + pneumonia	1
BSI + soft tissue infection (cheek, perianal abscess)	2
BSI + urethritis	1
Type of antibiotic treatment	
cefiderocol + fosfomycin + colistin ± meropenem	6
cefiderocol + amikacin + others antibiotics	4
cefiderocol + ceftazidime/avibactam + others	2
cefiderocol + tigecycline	2
cefiderocol + aztreonam + others	2
Duration of cefiderocol treatment days median (range)	15 (3–41)
ICU transfer	1
Outcome (*n* of patients)	13
Alive at 90 days	13/13
Relapse of MDR infection	2/13

^a^ In 2 episodes there was no site of infection in the presence of intestinal colonization.

**Table 4:** Episodes of cefiderocol use in children and adolescents with hematologic malignancies.

Ep *n*	Age Years	Sex	Hematol. Disease	Treatment Phase	Allogenic HSCT	Intestinal Colonization	Infection Definition	Isolated MDR-GN Bacteria	Type of Resistance	Cefiderocol Sensitivity	AST (Other)	MDR-GN Antimicrobial Combination	90-Day Outcome	Adverse Events
1	1	F	HLH	HSCT in 1st line	yes	*P. aeruginosa*	abdominal infection	*P. aeruginosa*	VIM	S	colistin amikacine	cefiderocol tigecyclin	Complete	no
2	3.5	M	ALL	1st line	no	*K. pneumoniae NDM*	bloodstream/urethritis	*K. pneumoniae*	NDM	S	colistin	cefiderocol meropenem	complete	no
3	8.7	M	ALL	2nd line relapse	no	*K. pneumoniae ESBL*	bloodstream	*K. pneumoniae*	ESBL	not available	colistin ceftazidime/avibactam	cefiderocol ceftaz/avib	complete	red wine urine s.
4	12.7	M	secondary AML	relapse post-HSCT	yes	no	bloodstream/soft tissue	*P. aeruginosa*	VIM	not available	colistin	cefiderocol meropenem colistin fosfomycin	complete	no
5	7.2	F	AML	2nd line	no	*P. aeruginosa VIM+*	bloodstream	*P. aeruginosa*	VIM	not available	colistin (I)	cefiderocol colistin levofloxacin	complete	no
6	17	M	ALL	HSCT in 2nd line relapse	yes	*P. aeruginosa VIM+*	urethritis	*P. aeruginosa*	VIM	not available	colistin	cefiderocol meropenem colistin fosfomycin	complete	no
7	17	F	AML	2nd line	no	no	bloodstream	*P. aeruginosa*	VIM	not available	colistin	cefiderocol colistin fosfomycin ceftaz/avib	complete	no
8	17.4	M	ALL	HSCT in 2nd line relapse	yes	*P. aeruginosa VIM+*	bloodstream/pneumonia		VIM	not available	colistin	cefiderocol meropenem colistin fosfomycin	complete	no
9	15.6	F	MDS	1st line	no	*P. aeruginosa VIM+*	bloodstream	*P. aeruginosa*	VIM	not available	colistin	cefiderocol meropenem colistin fosfomycin	complete	no
10	13.7	M	RCC	HSCT in 1st line	yes	*P. aeruginosa VIM+*	intestinal colonization T2	*P. aaeruginosa*	VIM	not available	not available	cefiderocol meropenem fosfomycin ceftaz/avib amikacin		
11	7.3	F	ALL	1st line	no	*P. aeruginosa*	bloodstream/soft tissue	*P. aeruginosa*	CRO no CPO	not available	colistin ceft/azi ceftoz/tazob aztreonam (I)	cefiderocol meropenem fosfomycin amikacin	complete	no
12	10.1	M	AML	HSCT in 1st line	yes	*P. aeruginosa VIM+*	intestinal colonization	*P. aeruginosa*	VIM	not available	not available	cefiderocol meropenem amikacin	complete	no
13	8.8	M	AML	1st line	no	*Enterobacter cloacae complex VIM+*	bloodstream	*K. pneumoniae*	VIM	S	aztreonam	cefiderocol fosfomycin amikacin aztreonam	complete	red wine urine s.
14	16	M	ALL	HSCT in 1st line	yes	no	pneumonia	*S. maltophilia*	-	-	not available	cefiderocol	complete	no
15	10	M	AML	1st line	no	*P. aeruginosa VIM+*	bloodstream	*P. aeruginosa*	VIM	not available	colistin	cefiderocol colistin fosfomycin	complete	no

HLH: hemophagocytic lymphohistiocytosis; ALL: acute lymphoblastic leukemia; AML: acute myeloid leukemia; MDS: myelodysplastic syndrome; RCC: refractory cytopenia of childhood; HSCT: hematopoietic stem cell transplantation; MDR-GN: multidrug-resistant Gram-negative; XDR extensively drug-resistant; NDM: New Delhi metallo-beta-lactamase; VIM: Verona integron-encoded metallo-beta-lactamase; CRO: carbapenem-resistant organisms; CPO: carbapenemase-producing organisms; AST other: antibiotic susceptibility testing for antibiotics other than cefiderocol.

## Data Availability

Data is contained within the manuscript.

## Abbreviations

The following abbreviations are used in this manuscript:

HSCT	Hematopoietic stem cell transplantation
MDR	Multidrug-resistant
IWG	Infection Working Group
AIEOP	Italian Association of Pediatric Hematology and Oncology
MBL	Metallo-beta lactamase
VIM	Verona integron-encoded metallo-beta-lactamase
NDM	New Delhi metallo-beta-lactamase
ECIL8	8th European Conference on Infections in Leukemia
IDSA	Infectious Disease Society of America
CR	Carbapenem-resistant
BSI	Bloodstream infection
ANC	Absolute neutrophil count
XDR	Extensively drug-resistant
CTCAE	Common terminology criteria for adverse events
ALL	Acute lymphoblastic leukemia
AML	Acute myeloid leukemia
CRO	Carbapenem-resistant organism
CPO	Carbapenemase-producing organism
ESBL	Extended spectrum beta lactamase
ICU	Intensive care unit
HLH	Hemophagocytic lymphohistiocytosis
MDS	Myelodysplastic syndrome
RCC	Refractory cytopenia of childhood
AE	Adverse event
SAE	Serious adverse event
AST	Antimicrobial susceptibility testing
